# Precision in Aortic Valve Therapy: Beyond Age, Sex, and Surgical Risk

**DOI:** 10.1016/j.jacadv.2026.103088

**Published:** 2026-08-10

**Authors:** Dritan Useini

**Affiliations:** Department of Cardiac Surgery, University Hospital Basel, University of Basel, Basel, Switzerland

I read with great interest the paper by Hendrianus et al^1^ published in a recent issue, providing valuable insights into comparative long-term outcomes of transcatheter aortic valve replacement (TAVR) vs surgical aortic valve replacement (SAVR). Evidence in this specific field has been scarce, and the findings of this study help fill an important gap.

Initially, eligibility for TAVR was primarily determined by surgical risk assessment, with scores such as the Society of Thoracic Surgeons Predicted Risk of Mortality score and EuroSCORE II serving as key decision-making tools.^2^ More recently, guideline recommendations^3^ have increasingly relied on age thresholds, resulting in a progressive lowering of the age at which TAVR is considered an acceptable or even preferred treatment option.

Yet both approaches share a common limitation. Neither the Society of Thoracic Surgeons Predicted Risk of Mortality score nor EuroSCORE II was developed to compare outcomes between TAVR and SAVR. These models were designed for surgical populations (encompassing gender, as well) and were subsequently extrapolated to TAVR patients despite substantial differences in patient characteristics, procedural risks, valve durability, and lifetime management considerations. Remarkably, despite the widespread adoption of TAVR and its expansion into lower-risk populations, no universally accepted risk model exists that reliably predicts which individual patient will derive greater long-term benefit from TAVR or SAVR.

The rationale for lowering age thresholds has largely been based on the compelling short-term advantages of TAVR. Lower early mortality, reduced procedural invasiveness, shorter hospital stays, and faster recovery have consistently favored the transcatheter approach. However, the assumption that these early benefits necessarily translate into durable long-term superiority is increasingly being challenged.

This debate has gained renewed momentum over the past year. Several contemporary meta-analyses, largely driven by randomized controlled trial data with extended follow-up,^4^^,^^5^ have consistently reported signals suggesting superior long-term outcomes after SAVR compared with TAVR, particularly with respect to overall survival. Although the magnitude and certainty of these findings vary across studies, the convergence of evidence has prompted a reappraisal of the current paradigm that favors progressive expansion of TAVR toward younger and lower-risk populations. These observations raise a fundamental question: Should treatment allocation continue to be guided primarily by age and procedural risk, or should it increasingly incorporate individualized predictions of long-term benefit?

Recent evidence has revealed a more complex picture. An updated meta-analysis of randomized trials demonstrated a clinically meaningful increase in 5-year all-cause mortality after TAVR compared with SAVR, accompanied by a high probability of increased stroke risk among low- and intermediate-risk patients.^4^ These findings suggest that procedural success and long-term effectiveness should not be considered synonymous.

At the same time, emerging evidence challenges the concept of a uniform class effect among transcatheter valves. A recent network meta-analysis of randomized trials demonstrated important differences between individual TAVR platforms, with some devices associated with increased all-cause and cardiovascular mortality compared with SAVR, whereas others achieved outcomes more comparable to surgery.^5^ These implications are particularly important as TAVR continues to move into younger populations.

The next revolution in aortic valve disease may not be a new prosthesis or a new procedure. It may be the ability to identify the right therapy for the right patient at the right time. In an era increasingly defined by precision medicine and artificial intelligence, individualized prediction—not age or surgical risk—should become the cornerstone of decision-making between TAVR and SAVR.
